# Is the evidence on the effectiveness of pay for performance schemes in healthcare changing? Evidence from a meta-regression analysis

**DOI:** 10.1186/s12913-021-06118-8

**Published:** 2021-02-24

**Authors:** Arezou Zaresani, Anthony Scott

**Affiliations:** 1 University of Manitoba, Institute for Labor Studies (IZA) and Tax and Transfer Policy Institute (TTPI), 15 Chancellors Circle, Fletcher Argue Building, Winnipeg, Manitoba Canada; 2 The University of Melbourne, Melbourne, Australia

**Keywords:** Financial incentives, Pay for performance (P4P), Value-based healthcare, Accountable care organization, Meta-regression analysis

## Abstract

**Background:**

This study investigated if the evidence on the success of the Pay for Performance (P4P) schemes in healthcare is changing as the schemes continue to evolve by updating a previous systematic review.

**Methods:**

A meta-regression analysis using 116 studies evaluating P4P schemes published between January 2010 to February 2018. The effects of the research design, incentive schemes, use of incentives, and the size of the payment to revenue ratio on the proportion of statically significant effects in each study were examined.

**Results:**

There was evidence of an increase in the range of countries adopting P4P schemes and weak evidence that the proportion of studies with statistically significant effects have increased. Factors hypothesized to influence the success of schemes have not changed. Studies evaluating P4P schemes which made payments for improvement over time, were associated with a lower proportion of statistically significant effects. There was weak evidence of a positive association between the incentives’ size and the proportion of statistically significant effects.

**Conclusion:**

The evidence on the effectiveness of P4P schemes is evolving slowly, with little evidence that lessons are being learned concerning the design and evaluation of P4P schemes.

**Supplementary Information:**

The online version contains supplementary material available at 10.1186/s12913-021-06118-8.

## Background

The use of Pay for Performance (P4P) schemes in healthcare has had its successes and failures. Using financial incentives targeted at healthcare providers to improve value-based healthcare provision is a key policy issue. Many governments and insurers use P4P schemes as a policy lever to change healthcare providers’ behavior to improve value for money in healthcare and make providers accountable. However, numerous literature reviews based on narrative syntheses of evidence have concluded that the evidence on P4P schemes’ impacts is both weak and heterogeneous (see, for instance [1–3]). The key reasons proposed for the weak evidence is that schemes have been either poorly designed (insufficient size of incentives, unintended consequences, unclear objectives, crowding out of intrinsic motivation, myopia, multi-tasking concerns, external validity, the scheme is voluntary, gaming), or poorly evaluated (poor study designs where causality cannot be inferred, no account of provider selection into or out of schemes, poor reporting of incentive design, poor reporting of parallel interventions such as performance feedback).

In this paper, we examined whether more recent studies provided improved evidence on the effectiveness of P4P schemes. One may expect that those designing and evaluating P4P schemes are improving how these schemes work and are being assessed. We updated the first meta-regression analysis of the effectiveness of P4P schemes [4] to investigate how more recent studies differed from the previous ones in the overall effects of the P4P schemes, the effects of study design on the reported effectiveness of the P4P schemes, and the effects of the size of incentives as a percentage of total revenue. A larger number of studies evaluating the effects of P4P helped us to increase the precision of the estimates of the effects in a meta-regression analysis and provided new evidence of changes in effects, study designs, and payment designs.

Scott et al. [4] found that an average of 56% of outcome measures per scheme was statistically significantly. Their findings suggested that studies with better study designs, such as Difference-in-Differences (DD) designs, had a lower chance of finding a statistically significant effect. They also provided preliminary evidence that the size of incentives as a proportion of revenue may not be associated with the chance of finding a statistically significantly outcome.

## Method

Our method was identical to that used by [4]. The same search strategy (databases and keywords) was extended from studies published between January 2010 and July 2015 (old studies) to studies published between August 2015 and February 2018 (new studies), and the same data were extracted from the new studies. Studies were included if they examined the effectiveness of a scheme on any type of outcome (e.g., costs, utilization, expenditures, quality of care, health outcomes and if incentives were targeted at individuals or groups of medical practitioners or hospitals).^1^

A vote-counting procedure was used to record the proportion of reported effect sizes that were statistically significant (at the 10% level) per study (noting if there were issues of the unit of analysis error and small sample size).^2^ Some studies examined a range of different outcome indicators, each with a reported effect size (i.e., usually the difference in an outcome indicator between an intervention and control group), while others examined the heterogeneity of effect sizes across time or sub-groups of the sample. Each separate effect size was counted when constructing the dependent variable (a proportion) for each study. For example, if a study reported an overall effect size and then analyzed the results separately by gender, then three effect sizes were counted.

A meta-regression analysis was conducted to examine factors associated with the proportion of statistically significant effects in each study, with each published paper as the unit of analysis. A generalized linear model with a logit link function (a fractional logit with a binomial distribution) was used because the dependent variable was a proportion with observations at both extremes of zero and one [7]. The error terms would not be independent if there was more than one study evaluating the same scheme, and so standard errors were clustered at the scheme level.

Our regression models included studies from [4] in addition to the new studies. Our estimates differed slightly from those estimated in [4] since we controlled for additional variables, including publication year, a dummy variable indicating studies from the new review, and the size of the P4P payments as a proportion of the total revenue. This was calculated from studies that reported both the size of the incentive amount and the provider’s total income or revenue. If there was a range of incentive amounts, we used the mid-point in the regression analysis.

Additional features of payment designs were extracted from the included papers and used in the regression models as independent variables, to the extent that they were reported in the papers reviewed. The additional features included whether the scheme included incentives for improvements in both quality and cost or quality alone (an important design innovation as used in shared savings schemes in the US’s Accountable Care Organizations), whether the scheme rewarded for quality improvement over time rather than meeting a threshold at a specific point in time, and how incentives were used by those receiving the incentive payments (physician income, discretionary use, or specific use such as quality improvement initiatives). We also included a categorical variable for the study design used: Difference-in-Difference (DD), Interrupted Time Series (ITS), Randomized Controlled Trial (RCT), and Before-After with regression (BA) as the reference group. Studies with no control groups and studies which did not adjust for covariates were excluded. We further included a dummy variable for schemes in the US where arguably most experimentation has occurred to date, and whether the scheme was in a hospital or primary care setting.

## Results

A total of 448 new papers were found, of which 302 papers were included after screening the title and abstract. Of these, 163 were empirical studies, and full-text screening identified 37 new studies that were eligible for inclusion [4].

Table 1 presents a summary of the new studies by the country of the evaluated P4P scheme.^3^ The new studies evaluated 23 different schemes across 12 countries, including schemes in new countries (Afghanistan, Kenya, Sweden, and Tanzania). This finding reflects an increase in the proportion of P4P schemes from countries other than the US (from 23% versus 56%) compared to the studies in [4]. There was also a slightly lower proportion of schemes in new studies, which are conducted in hospitals (25% versus 29.5%).

**Table 1:** Number of new (between 2015 and 2018) studies and schemes added to the review by country and setting

Country	Hospital	Multispecialty or Primary care physician	Number of studies	Number of schemes
Afghanistan	0	1	1	1
Australia	1	0	1	1
Canada	1	6	7	7
China	0	1	1	1
France	0	3	3	1
Kenya	0	1	1	1
Rwanda	0	1	1	1
Sweden	0	1	1	1
Taiwan	5	5	10	2
Tanzania	1	2	3	1
UK	0	2	2	1
US	2	4	6	6
Total	10	27	37	23

Table 2 summarizes the overall effects of the schemes evaluated in new studies. The 37 studies across 23 schemes reported on 620 effect sizes (average of 26.95 per scheme and 16.76 per study), of which 53% were statistically significant (53% per scheme; 60% per study). This compared to 46, 56, and 54%, respectively, from [4], suggested a slight increase in the proportion of significant effects, but an overall mixed effects of P4P schemes.

**Table 2:** Summary of overall effects

	Mean	Median	Min-Max
A: By scheme (n= 23)			
Number of significant effect sizes	14.17	7.00	0–73
Number of effect sizes	26.95	18.00	1–108
Proportion of significant effect sizes	0.53		
B: By study (n= 37)			
Number of significant effect sizes	8.81	6.00	0–38
Number of effect sizes	16.76	12.00	1–108
Proportion of significant effect sizes	0.60		
	**Total**	**Statistically significant**	**Proportion**
**Total reported effect sizes**	**620**	**326**	**0.53**

Table 3 suggests that the P4P schemes had mixed effects on the range of outcomes considered. Table 4 shows that countries with the highest proportion of statistically significant effects are Kenya (82%), France (81%), Taiwan (75%), and Tanzania (73%). Only one study, in Afghanistan, showed no effects.

**Table 3:** Mean proportion of statistically significant effect sizes by the scheme

Schemes	Proportion of significant effect sizes	Number of effect sizes	Number of studies
^a^Afghanistan P4P	0	7	1
CAPI-ROSP	0.82	37	3
^a^Chronic disease P4P	0.20	35	1
^a^Community-based psychiatric care P4P	1	6	1
^a^Highmark’s Quality Blue (QB) in Pennsylvania	1	1	1
^a^Improve hospital discharge follow-ups P4P	0	5	1
^a^Medicaid	0.18	28	1
^a^Mental health P4P	1	24	1
^a^P4P for Antibiotics	0.50	6	1
P4P for Diabetes	0.74	98	9
P4P for Hepatitis	0.75	8	2
^a^P4P for Immunization	0.36	28	1
P4P for Malaria	0.82	11	1
^a^P4P for Maternal care	0.73	67	3
^a^P4P for drug prescription	0.50	24	1
^a^Partners for Kids	0.67	21	1
^a^Pay for results P4P	0.60	63	1
^a^Physician Integrated Network (PIN)	0.67	3	1
Quality and Outcomes Framework	0.33	6	2
^a^Queensland P4P	0.25	4	1
Rwanda P4P	0.19	108	1
^a^Spontaneous breathing (SBTs) P4P	0.50	18	1
^a^Low-Density lipoprotein Cholesterol (LDL-C)	0.33	12	1

Note: ^a^ denotes the new schemes evaluated in the new studies

**Table 4:** Mean proportion of significant effect sizes by country

Country	Proportion of significant effect sizes	Number of effects sizes	Number of studies	Number and schemes
Afghanistan	0	7	1	1
Australia	0.25	4	1	1
Canada	0.57	148	7	7
China	0.50	24	1	1
France	0.81	37	3	1
Kenya	0.82	11	1	1
Rwanda	0.19	108	1	1
Sweden	0.50	6	1	1
Taiwan	0.75	94	10	2
Tanzania	0.73	67	3	1
UK	0.33	6	2	1
US	0.40	108	6	6

### Meta-regression analysis

Table 5 presents the meta-regression analysis using 116 studies evaluating P4P schemes published between January 2010 to February 2018. A full set of descriptive statistics is presented in Additional file 2 Appendix B. The three models in Table 5 had different sample sizes because of the covariates in each model were different.

**Table 5:** Factors associated with the proportion of significant effect sizes

	Model 1		Model 2		Model 3	
	Average Marginal Effect (AME)	Sample mean	Average Marginal Effect (AME)	Sample mean	Average Marginal Effect (AME)	Sample mean
New studies (August 2015-Feb 2018)	0.11 (0.13)	0.32 (0.47)	0.06 (0.17)	0.30 (0.46)	0.05 (0.16)	0.26 (0.44)
**Research design**						
Difference in Differences (DID)	−0.20** (0.08)	0.43 (0.50)	−0.20** (0.10)	0.44 (0.50)	− 0.24** (0.10)	0.52 (0.51)
Interrupted Time Series (ITS)	−0.09 (0.10)	0.12 (0.33)	−0.11 (0.11)	0.13 (0.34)	−0.67*** (0.08)	0.09 (0.30)
Randomized Control Trial (RCT)	−0.19* (0.10)	0.41 (0.49)	−0.20* (0.11)	0.10 (0.30)	−0.34*** (0.12)	0.14 (0.35)
**Country**						
US	−0.11* (0.07)	0.41 (0.49)	−0.13* (0.07)	0.41 (0.49)	0.08 (0.11)	0.40 (0.50)
**Setting**						
Hospital	0.06 (0.06)	0.30 (0.46)	0.05 (0.07)	0.32 (0.47)	−0.24** (0.09)	0.33 (0.48)
**Incentive Schemes**						
Cost and quality	0.11* (0.06)	0.25 (0.43)	0.13* (0.08)	0.25 (0.44)	−0.34*** (0.08)	0.21 (0.41)
**Rewards for improvement**	−0.12* (0.07)	0.36 (0.48)	−0.12* (0.07)	0.38 (0.49)	−0.09 (0.06)	0.36 (0.49)
**Use of incentives**						
Discretionary use			0.01 (0.09)	0.55 (0.50)		
Special use			0.08 (0.12)	0.06 (0.25)		
Size of payment to revenue ratio (%)					0.01* (0.00)	11.53 (9.18)
Publication year fixed effects	Yes		Yes		Yes	
Number of observations	116		109		42	
Number of clusters	62		57		25	
Average number of observations per cluster	1.9		1.9		1.7	
(Min-Max)	(1–15)		(1–14)		(1–4)	
Pseudo-Log	−59.91		−55.66		−16.99	
AIC	1.33		1.37		1.57	
BIC	− 408.42		−365.18		− 84.57	

Note: The omitted reference group for “Research Design” is before and after designs, controlled before and after, and case-control studies. The omitted reference group for “Country” is all other countries. The omitted reference group for “Setting” is primary care. The Omitted reference group for “Incentive schemes” is to pay for performance only. The omitted reference group for “Rewards for improvement” is not rewarding for improvement. The Omitted reference group for “Use of incentives” is using incentives for physician income. The standard errors are clustered in the scheme level and are in the parenthesis

∗*p* < 0.10, ∗ ∗ *p* < 0.05, ∗ ∗ ∗*p* < 0.01

*AIC* Akaike information criterion

*BIC* Bayesian information criterion

The first model suggested that the new studies were 11 percentage points more likely to report statistically significant effects, although the estimated coefficient itself was not statistically significant. Studies with DD and RCT designs were respectively 20 and 19 percentage points less likely to report a statistically significant effects compared with the studies with BA with control designs. Studies with ITS design were not statistically different compared with the BA studies. The role of study design in explaining the proportion of statistically significant effects was weaker compared with [4], which reported 24 and 25 percentage points differences for DD and RCT designs, respectively. The proportion of different study designs in the new compared to old review were similar, suggesting that the difference in the effect of study design might be due to the studies with RCT, ITS, and DD designs in the new review having lower proportions of statistically significant effects relative to the studies with BA designs.

Studies evaluating P4P schemes that used incentives for improving both costs and quality (compared to the quality alone) were 11 percentage points more likely to report statistically significant effect sizes. The coefficient was slightly larger than in [4] and statistically significant at the 10% level. This might be due to overall weaker effects from the new studies since none of the new studies examined this type of payment design. Studies evaluating P4P schemes, which rewarded for improvement over time, led to a lower proportion of statistically significant effect sizes.

**Fig. 1:**
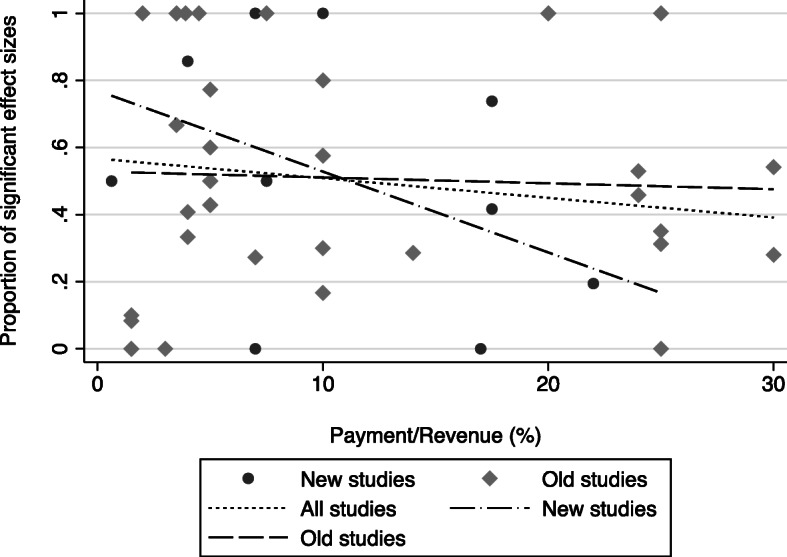
Relationship between the relative size of incentive payments to revenue and portion of significant effect sizes

Scott et al. [4] found that the use of payments for specific purposes (such as payments for investment quality improvement rather than physician income) was associated with a 24 percentage points increase in the proportion of statistically significant effect sizes, but this has fallen to 8 percentage points (Model 2). Of the 23 new schemes included in the review, eight schemes included information on the size of the incentive payments relative to total revenue, ranging from 0.05 to 28% (see Table 6 for more details). Figure 1 shows the unadjusted correlation between the size of payments and the proportion of statistically significant effect sizes, and includes regression lines for old, new, and all studies combined. Studies from the new review showed a stronger negative relationship between the payment size and effectiveness, based on only 11 studies (Table 6). After controlling for other factors in the main regression in Table 5, the results suggested a small positive association between the size of incentives and the proportion of effect sizes statistically significant at the 10% level (Model 3).

**Table 6:** Relative size of incentive payments to revenue

Scheme, study	Country	Payment/Revenue (%)
Spontaneous breathing trials (SBTs), [8]	US	7.5
CAPI-ROSP, [9–11]	France	4–7
Rwanda P4P, [12]	Rwanda	22
P4P for Maternal care, [13–15]	Tanzania	10–25
P4P for Antibiotics, [16]	Sweden	0.05–1.2
Quality and Outcomes Framework, [17]	UK	25
Afghanistan P4P, [18]	Afghanistan	6–28

## Discussion

We examined if more recent studies changed the conclusions of the first meta-analysis of P4P literature as P4P schemes continue to evolve. This evolution could be better scheme design, or that the same designs are being used in more settings where our results suggested the latter. Thirty-seven recent studies over a two-year period were added to the existing 80 studies reported in a previous review. The RCT and DD study designs reduced the proportion of statistically significant effects by a lower amount than the previous review. The range of countries in which P4P is being evaluated had increased, with some weak evidence that the proportion of studies with statistically significant effects had increased. The effectiveness of P4P remained mixed overall.

There may be unobserved factors within each scheme associated with both the size of incentive and the likelihood of a statistically significant effects. This variable could be measured with error as some studies present a range rather than an average, of which we took the midpoint.

Despite mounting evidence on the effectiveness of P4P schemes, differences in payment design that were able to be extracted from the studies seemed to play a minor role. However, this meta-analysis masked much heterogeneity in the context, design, and implementation of schemes that were unlikely to be reported in the published studies. Schemes included various diseases, outcome measures, and payment designs, including schemes where the additional funding was used rather than re-allocating existing funding. The choice of outcome indicators may influence the effectiveness of each scheme as some may be more costly for physicians to change than others, while others, such as health outcomes, may be more dependent on patient’s behavior change.

Clustering by the scheme may have accounted for some of this unobserved heterogeneity within schemes. There may also be interactions between different features of payment design, though to examine these would require more clear hypotheses about such interactions and more detailed reporting of payment designs. Data extraction was limited to published studies that vary how they reported the payment designs and study designs. There needs to be improved and more standardized reporting in the literature to enable more general lessons to be learned from P4P schemes that can guide their successful design and implementation [19].

## Conclusion

The factors influencing the success of schemes have not changed remarkably. When assessing the studies, none provided any justification for the particular incentive design used, and none stated they were attempting to improve the way incentives were designed compared to previous schemes. This may be because many schemes were designed within a specific local context, and so were constrained in the design they could use and the scheme’s objectives. This could also be because they were constrained in the indicators they use and can collect and report, suggesting that improvements in information and data on quality occur only very slowly over time.

There should be no expectation that incentive designs should necessarily be becoming more complex over time, as this can lead to gaming and ignores the existence of already strong altruistic motives to improve the quality of care. Incentive schemes were also a result of negotiation between providers and payors, and this presented constraints in how incentives were designed. Providers attempted to extract maximum additional income for minimum behavior change, and payors try and achieve the opposite. This could mean that the resulting schemes were relatively weak in terms of incentives’ strength and size. The value of some schemes to policymakers could be more about the increased accountability they seemed to provide, rather than changing behavior and improving value-based healthcare itself. Similarly, although providers were interested in enhancing quality, their preference for self-regulation and autonomy and protection of income, meant that top-down comparisons of performance and potential reductions in earnings were likely to have been unwelcome. Though payment models must support and not hinder value-based healthcare provision, they remained ‘one size fits all’ fairly blunt instruments that needed to be supplemented with other behavior change interventions.

### Article summary

#### Strength and limitations

- This study updated the first meta-regression analysis of the effectiveness of Pay-for-Performance (P4P) schemes to examine if the factors affecting P4P schemes’ success had changed over time as these schemes evolved.
- Data extraction was limited to published studies that varied in how they reported the payment and the study designs.
- This meta-analysis masked much of heterogeneity in the context, design, and implementation of schemes that were unlikely to be reported in the published studies.

## Supplementary Information

**Additional file 1.** Appendix A. Metadata collected by the authors and [4] ([20–43]).

**Additional file 2.** Appendix B. Metadata collected by the authors of [4] ([44–75]).

## Data Availability

The extracted data and the statistical analysis code are available from the corresponding author upon reasonable request.

## Abbreviations

P4P: Pay for performance
DD: Difference in Differences
RCT: Randomized Control Trial
BA: Before-After
ITS: Interrupted Time Series
UK: United Kingdom
US: United States
QOF: Quality and Outcomes Framework
SBT: Spontaneous breathing trials
ACO: Accountable Care Organizations
GP: General practitioner
CAPI-ROSP: France’s national P4P scheme for primary care physicians
QB: Quality Blue
LDL-C: Low-density lipoprotein cholesterol
PIN: Physician Integrated Network
AIC: Akaike information criterion
BIC: Bayesian information criterion
ICU: Intensive Care Unit
MAPD: Medicare Advantage Prescription Drug
HVBP: Medicare Value-Based Purchasing
MHIP: Mental Health Information Program
QIDS: Quality Improvement Demonstration Study

## Footnotes

1 ﻿We searched “*PubMed”* and “*EconLit”* electronic databases for journal articles with the keywords of *“value-based purchasing,” “pay for performance,”* and *“accountable care organisations*,” focusing on studies evaluating a P4P scheme or ﻿schemes rewarding for cost reductions. We restricted our search to studies in English from high-, middle and low-income countries, and we excluded studies using qualitative data, reviews of the literature, editorials, and opinion pieces, and studies with no control group, and studies which did not adjust for confounders. ﻿We included studies in our review if they examined the impact of schemes on any type of outcome (e.g., costs, utilization, expenditures, quality of care, health outcomes), and if the incentives were targeted at individual or groups of medical practitioners or hospitals. We identified 37 new studies.

2 Vote-counting is recommended where the outcomes of studies are too heterogeneous to pool the data together [5]. For an overview of reviews using vote-counting, see [6].

3 More details of the new studies are provided in Appendix A. A summary of the pooled new and old studies is presented in Table B.1 in Appendix B.
