# Supplementary material for: Is the evidence on the effectiveness of pay for performance schemes in healthcare changing? Evidence from a meta-regression analysis
Source: BMC Health Serv Res. 2021 Feb 24;21:175. doi: 10.1186/s12913-021-06118-8 (PMC7905606; doi:10.1186/s12913-021-06118-8)
Supplement: Supplementary file 1 — Additional file 1. Appendix A. Metadata collected by the authors and [4] ([20–43]). [file 12913_2021_6118_MOESM1_ESM.pdf]

Appendix A: Summary of the recent studies (published August 2015 – February 2018)

Six studies evaluated six schemes in the US, three oof which were focused on Medicaid schemes within Accountable Care Organizations (ACO), and three were stand-alone schemes in hospitals and one for primary care physicians. Of the Medicaid schemes, [20] focused on primary care physicians in a pediatrics ACO. [21] studied immunization within all 19 states running Medicaid P4P programs. The third study examined three state-based Medicaid P4P schemes but focused on a wide range of performance measures.

Of the remaining three studies, [8] examined care provided in ICUs in three hospitals Pennsylvania, and [22] examined bloodstream infections as part of a larger P4P scheme in 52 hospitals across 29 counties in Pennsylvania. [23] focused on reducing levels of low-density lipoprotein cholesterol using a Randomized Control Trial (RCT) in the northeastern US using both patient and provider incentives.

Seven studies evaluated seven schemes in Canada. Three studies examined three schemes in Ontario, two examined two schemes in British Columbia, one study examined the Physician Integrated Network scheme in Manitoba, and one study evaluated a scheme for diabetes in New Brunswick. Of the three schemes in Ontario, [24] examined a scheme to reduce the length of stay in emergency departments. [25] examined the effect of a new billing code for physicians for follow-up within 14 days of hospital discharge for medical and surgical patients across Ontario. [26] examined the impact of an increase in service fees plus an annual payment per patient for psychiatrists providing outpatient care within 30 days after discharge from a psychiatric hospital and 180 days after a suicide attempt. [27] evaluated a P4P scheme for chronic disease management

in primary care in British Columbia, which introduced a new annual capitation payment per patient within the fee schedule, on top of the existing fee for service payments. [28] focused on a scheme strengthening the existing fee-for-service system with additional fees for counselling and care coordination and preparing a management plan in mental healthcare in British Colombia. [29] evaluated the effect of a scheme across 12 primary care clinics in Manitoba on childhood immunization. [30] evaluated a scheme which added annual per patient capitation payment for diabetes patients.

Three studies evaluated France's national P4P scheme for primary care physicians (CAPI-ROSP).[4] Each of these studies focused on different outcomes. The payments were made to attain the targets defined by fixed percentages of the population receiving each service. [10] focused on benzodiazepine prescribing, [9] focused on breast cancer screening, and [11] focused on cervical screening.

Two studies evaluated the Quality and Outcomes Framework (QOF) for GPs in the UK. [17] examine long-terms trends on the effect of the QOF on mortality rates for chronic disease, and [31] examined the risk of nursing home admissions for dementia. A study from Australia evaluated a scheme examining access to the first dialysis across the public hospital system in Queensland [32]. [15] examined the effect of a scheme in primary care across eight municipalities in Sweden to encourage appropriate antibiotic prescribing for young children.

---

[4] CAPI was the precursor scheme and was voluntary, and then extended nationwide and renamed ROSP using an expanded set of indicators.

There were ten new studies evaluating two schemes in Taiwan, focusing on diabetes and hepatitis. The scheme for Diabetes was introduced in 2001 as part of the changes to the National Health Insurance, which introduced new fees under the existing fee-for-service scheme and per patient (capitation) fees for case management. For the diabetes studies, [33] used a subgroup of diabetic patients, namely those also with hypertension, and focused on the number of visits and measures of continuity of care between 2004 and 2007. [34] also examined this group of patients using very similar methods and data over the same period but used the rates of examinations and visits as the outcome variable. [35] examined the cost-effectiveness for the same group of patients using data from 2007 to 2012. [36] examined the effects on continuity of care and mortality using data from 1997 to 2009. [37] focused on mortality between 2001 and 2009 while [38] focused on macro-vascular complications between 2007 and 2012. [39] examined a change in the design of the scheme that added outcome measures (change in HbA1c) in 2006 to existing process measures. The scheme for Hepatitis was introduced in 2010, which used new case management fees and payments for follow up visits. Using data from 2009 to 2011, [40] focused on visits, examinations and tests as outcome variables. [41] focused on the risk of hospital admissions and the development of liver cirrhosis from 2010 to 2013.[5]

Six studies were from low-and middle-income countries, five of which focused on maternal and child health services. In Tanzania, three studies evaluated the effect of a P4P scheme for maternal and child health services on utilization, quality, costs [13, 14, 42]. A study from Afghanistan also

[5] An earlier version of [4] was included in [4]. We included the latest version and dropped it from the sample of older studies in our analysis.

examined maternal and child health services [18]. [12] examined multi-tasking issues in a maternal and child health services P4P scheme in Rwanda [2]. [43] evaluated a P4P to increase diagnostic testing and appropriate prescribing in the management of malaria in Kenya.
