# Supplementary material for: Is the evidence on the effectiveness of pay for performance schemes in healthcare changing? Evidence from a meta-regression analysis
Source: BMC Health Serv Res. 2021 Feb 24;21:175. doi: 10.1186/s12913-021-06118-8 (PMC7905606; doi:10.1186/s12913-021-06118-8)
Supplement: Supplementary file 2 — Additional file 2. Appendix B. Metadata collected by the authors of [4] ([44–75]). [file 12913_2021_6118_MOESM2_ESM.pdf]

Appendix B: Tables from the pooled new studies and studies from [4]

Table B.1: Number of studies and schemes included in the review by country and setting

Country	Hospital	Multi-specialty or Primary care physician	Number of studies	Number and schemes
Afghanistan	0	1	1	1
Australia	1	2	3	3
Canada	2	8	10	9
China	0	3	3	2
France	0	4	4	1
Italy	1	1	2	2
Kenya	0	1	1	1
Philippines	1	0	1	1
Rwanda	0	2	2	1
Sweden	0	1	1	1
Taiwan	8	11	19	4
Tanzania	1	2	3	1
UK	5	13	18	5
US	16	32	48	50
Total	35	81	116	62

Table B.2: Summary of overall effects

	**Mean**	**Median**	**Min-Max**
A: By scheme (n=62)			
Number of significant effect sizes	14.72	5.00	0-156
Number of effect sizes	30.54	10.00	1-332
Proportion of significant effect sizes	0.57		
B: By study (n=116)			
Number of significant effect sizes	7.87	6.00	0-68
Number of effect sizes	16.33	12.00	1-120
Proportion of significant effect sizes	0.56		
	**Total**	**Statistically significant**	**Proportion**
**Reported effect sizes**	1,894	913	0.48

Table B.3: Mean proportion of significant effect sizes by scheme

**Scheme**	**Proportion of significant effect sizes**	**Number of effect sizes**	**Number of studies**
Advancing Quality	0.35	60	3
Afghanistan P4P	0	7	1
Alabama Managed Care Organization	0.33	9	1
Alternative Quality Contract	0.47	332	8
BSBC (Michigan) Physician Group Incentive Program	0.78	22	1
Best Practice Tariff	0.49	41	2
Breast Cancer P4P	1	3	1
Bronx CHAMPION	0.60	5	1
CAPI-ROSP	0.69	48	4
Chronic disease P4P	0.2	35	1
Clinical directed enhanced services	0.83	6	1

Community-based psychiatric care P4P	1	6	1
Diabetes Care Project	0.32	34	1
ED P4P	1	2	1
Emilia-Romagna P4P	0.50	2	1
Fairview Health Services	0.31	39	1
Health Plan in Hawaii	1	4	1
Highmark's Quality Blue (QB) in Pennsylvania	1	1	1
Houston/Harris County Community Health Program	0.67	3	1
Hudson Health Plan - diabetes	0	12	1
Hudson Health Plan - immunization	1	2	1
Improve hospital discharge follow-ups P4P	0	5	1
Kaiser Permanent Northern California	1	2	1
Lazio DRG P4P	1	4	1
Medicaid	0.18	28	1
Medicaid Mass Health	0	2	1
Medicare Advantage Prescription Drug Plan (MAPD)	0.29	17	1
Medicare Dialysis payment Reform	1	1	1
Medicare Hospital Value-Based Purchasing (HVBP)	0	6	1
Medicare Never Events	0.57	7	2
Medicare Pioneer ACOs	0.50	109	3
Medicare Premier Hospital Quality Incentive Demonstration	0.10	49	5
Mental health P4P	1	24	1
Mental Health Integration Program (MHIP)	1	1	1
NHS stop smoking services	0.33	6	1
Ningxia scheme	0.41	49	2
Ontario P4P	0.5	8	2
P4P for Antibiotics	0.5	6	1

P4P for Diabetes	0.74	163	15
P4P for Hepatitis	0.75	8	2
P4P for Immunization	0.36	28	1
P4P for Malaria	0.82	11	1
P4P for Maternal care	0.73	67	3
P4P for Tuberculosis	1	2	2
P4P for drug prescription	0.50	24	1
Pacific Care /IHA	0.28	54	1
Partners Health Care, Inc.	1	1	1
Partners for Kids	0.67	21	1
Pay for results P4P	0.60	63	1
Physician Group Practice Demonstration	0.62	122	3
Physician Integrated Network (PIN)	0.67	3	1
Practice Incentives Program	0.50	4	1
Primary Care Information Project	0.60	20	2
Quality and Outcomes Framework	0.39	119	11
Quality Improvement Demonstration Study (QIDS)	0.50	4	1
Queensland P4P	0.25	4	1
RWF-AHRG Health Promotion	1	1	1
Rwanda P4P	0.20	115	2
Spontaneous breathing (SBTs) P4P	0.50	18	1
Veterans Affairs networks	0.58	12	1
eHeart (pilot)	0.38	21	1
Low-Density Lipoprotein Cholesterol (LDL-C)	0.33	12	1

Table B.4: Mean proportion of significant effect sizes by country

**Country**	**Portion of significant effect sizes**	**Number of effect sizes**	**Number of studies**	**Number of schemes**
Afghanistan	0	7	1	1

Australia	0.33	42	3	3
Canada	0.58	158	10	9
China	0.59	143	12	6
France	0.69	48	4	1
Italy	0.83	6	2	2
Kenya	0.82	11	1	1
Philippines	0.50	4	1	1
Rwanda	0.20	115	2	1
Sweden	0.50	6	1	1
Taiwan	0.75	94	10	0
Tanzania	0.73	67	3	1
UK	0.40	232	18	5
US	0.45	961	48	31

Table B.5: Relative size of incentive payments to revenue

**Scheme, Study**	**Country**	**Payment/Revenue (%)**
P4P for Antibiotics, [16]	Sweden	0.05-1.2
Medicare Hospital Value-Based Purchasing, [44]	US	1-2
Medicare Premier Hospital Quality Incentive Demonstration, [45, 46]	US	1-2
Medicare Dialysis Payment Reform, [47]	US	2
Breast Cancer P4P, [48]	Taiwan	2-7
Health Plan in Hawaii, [49][50]	US	3.5
Houston/Harris County Community Health Program, [51]	US	3-4
Partners Health Care, [52, 53]	US	3-4.8
Ontario P4P, [54, 55]	Canada	3-10
Advancing Quality Initiative, [10, 11]	UK	4
CAPI-ROSP, [56]	France	4-7
Bronx CHAMPION, [57, 58]	US	5
Primary Care Information Project, [59]	US	5
Quality Improvement Demonstration Study, [60]	Philippines	5
Blue Cross Blue Shield of Michigan’s Physician Group Incentive Program, [18]	US	5-10
Afghanistan P4P, [61]	Afghanistan	6-28
Contract for Improving Individual Practices, [8]	France	7
Spontaneous breathing trials (SBTs), [62–64]	US	7.5
Alternative Quality Contract, [12–14]	US	10
P4P for Maternal care, [65]	Tanzania	10-25
Rwanda P4P, [66]	Rwanda	14
Hudson Medicaid Health Plan, [67, 68]	US	15-25
Best Practice Tariff, [69]	UK	24
Mental Health Integration Program, [17, 70–72]	US	25
Quality and Outcomes Framework, [73]	UK	25
Shandong scheme, [74, 75]	China	Up to 20%
Ningxia scheme, [2]	China	Up to 30%
